# Plastome Evolution in *Viburnum* (Adoxaceae): Comparative Genomics Reveals Hypervariable Markers and Relaxed Selection on Protein Import Genes

**DOI:** 10.3390/genes17020196

**Published:** 2026-02-06

**Authors:** Lanruo Mou, Qiang Zhang, Bingyue Zhu, Chao Shi, Jing Yang

**Affiliations:** 1 College of Biological Engineering, Qingdao University of Science and Technology, Qingdao 266042, China; moulanruo@163.com (L.M.); zhangqiang@qust.edu.cn (Q.Z.);; 2 College of Chemical Engineering, Qingdao University of Science and Technology, Qingdao 266042, China

**Keywords:** chloroplast genome, comparative genomics, Dipsacales, molecular markers, *Ka*/*Ks*, phylogenomics

## Abstract

Background: *Viburnum* (Adoxaceae) is a species-rich woody genus whose taxonomy is complicated by morphological convergence and hybridization. Methods: We assembled complete plastomes of eight species representing five sections and analyzed their structural variation, sequence divergence, and molecular evolution. Results: All plastomes displayed the conserved quadripartite structure typical of angiosperms, with limited size variation attributable primarily to intergenic spacer-length polymorphisms. Sequence divergence was unevenly distributed, with single-copy regions exhibiting substantially higher nucleotide diversity than inverted repeat regions. We identified multiple hypervariable intergenic spacers such as the region *trnK-UUU–rps16*, suitable as molecular markers for population genetics and species identification. Selection pressure analysis revealed that while most protein-coding genes evolved under strong purifying selection, three genes involved in fatty acid biosynthesis and protein import—*accD*, *ycf1*, and *ycf2*—showed significantly relaxed constraints, suggesting ongoing functional divergence. Phylogenetic analysis recovered well-supported relationships consistent with previous classifications, while clarifying the positions of *Viburnum amplificatum* and *Viburnum tinus*. Conclusions: These findings provide molecular resources for *Viburnum* systematics and offer insights into the evolutionary dynamics of plastome genes with non-photosynthetic functions.

## 1. Introduction

The genus *Viburnum* L. (Adoxaceae, Dipsacales) comprises approximately 230 species of shrubs and small trees distributed predominantly across temperate and subtropical regions of the Northern Hemisphere [1]. Asia represents the primary center of diversity, with China alone harboring 96 species—the highest national diversity globally [2]. The genus exhibits remarkable ecological amplitude, occupying habitats ranging from temperate deciduous forests to subtropical cloud forests and Mediterranean shrublands, and many species are cultivated as ornamentals or used in traditional medicine [3,4]. Despite intensive study, species delimitation within *Viburnum* remains challenging. Morphological characters traditionally used for classification—including endocarp architecture, inflorescence type, and trichome morphology—show extensive homoplasy, and frequent hybridization both in nature and cultivation further obscures species boundaries [5]. Molecular phylogenetic studies have substantially improved understanding of relationships within the genus, revealing that several traditionally recognized sections are not monophyletic and necessitating taxonomic revision [1,6]. Despite these advances, the integration of plastome-scale genomic data with evolutionary and functional interpretations remains limited for *Viburnum*, particularly at the inter-sectional level.

Chloroplast genomes have proven particularly valuable for resolving relationships within *Viburnum*. The relatively slow evolutionary rate of plastomes, combined with their uniparental inheritance and lack of recombination, makes them well-suited for phylogenetic reconstruction at various taxonomic levels [7]. Previous studies have sequenced plastomes from numerous *Viburnum* species, establishing a robust phylogenetic framework and resolving major clades within the genes [6,8]. However, existing studies have largely focused on tree topology, whereas comparative analyses of plastome evolution—especially those integrating structural variation and selection pressures on protein-coding genes—remain scarce in *Viburnum*. In particular, it remains unclear whether plastome genes exhibit lineage- or section-specific patterns of molecular evolution, and how such patterns may relate to functional divergence or historical biogeography within the genus. Although plastome gene content and organization are broadly conserved across angiosperms, individual genes vary considerably in their evolutionary rates [7]. Genes encoding components of the photosynthetic apparatus typically evolve under strong purifying selection, reflecting their essential metabolic functions. By contrast, genes involved in other processes—including fatty acid synthesis (*accD*) and protein import (*ycf1*, *ycf2*)—often show accelerated evolution, potentially reflecting functional divergence or relaxed constraint [9,10]. Characterizing these patterns across diverse lineages can reveal the evolutionary forces shaping plastome architecture.

In this study, we assembled and analyzed complete plastomes from eight *Viburnum* species representing five sections and spanning both Old World and New World distributions. By integrating plastome structural comparisons, molecular evolutionary analyses, and phylogenetic inference, we aimed to address three key questions: first, to systematically characterize structural variation in *Viburnum* plastomes and identify hypervariable regions suitable for molecular marker development; second, to assess selection pressures on protein-coding genes and identify candidates for functional divergence; and third, to evaluate phylogenetic relationships and their congruence with existing classifications. Through these analyses, this work not only supplements the chloroplast genome database of the genus *Viburnum* and evolutionary research, but also offers new insights into the evolutionary dynamics of plastid genes within a morphologically complex and taxonomically challenging genus.

## 2. Materials and Methods

### 2.1. Plastome Assembly and Annotation

Raw sequencing data for eight *Viburnum* species representing major lineages and geographic regions: *Viburnum molle* Michx. and *Viburnum dentatum* L. (section Porphyrotinus, eastern North America), *Viburnum acerifolium* L. (section Succotinus, eastern North America), *Viburnum lentago* L. (section Valvatotinus, North America), *Viburnum amplificatum* Kern (section Crenotinus, Southeast Asia), *Viburnum grandiflorum* Wall. ex DC. (section Solenotinus, Himalaya), *Viburnum plicatum* Thunb. (section Tomentosa, East Asia), and *Viburnum tinus* L. (section Tinus, Mediterranean) were obtained from the NCBI BioProject database (https://www.ncbi.nlm.nih.gov/ (accessed on1 February 2026)). Raw reads were processed using Trimmomatic v0.39 [11]. Quality-filtered reads were assembled into complete plastomes using GetOrganelle v1.7.7 [12] with parameters optimized for embryophyte plastomes. This pipeline employs a reference-guided extension algorithm that iteratively maps reads to seed sequences, followed by de novo assembly of mapped reads using SPAdes (v.4.2.0). Assembly graphs were visualized and inspected using Bandage v0.8.1 [13] to verify complete circular assembly and resolve any ambiguities at inverted repeat boundaries. For each species, we confirmed that the assembly graph showed the expected structure with two alternative paths through the inverted repeats. Final assemblies achieved mean sequencing depths ranging from 312× to 587× across species.

Plastome annotation was performed using GeSeq v2.03 [14] with *Viburnum japonicum* (MH036493) and *Viburnum farreri* (NC_056112) as references. The annotation pipeline employed BLAT for protein-coding genes, HMMER for rRNAs, and ARAGORN and tRNA scan-SE for tRNAs. All annotations were manually verified and refined in Geneious Prime 2023.1 (Biomatters, Auckland, New Zealand). Start and stop codon positions were checked against amino acid alignments of orthologous genes from related species, and any apparent frameshifts or premature stop codons were examined for possible annotation errors or pseudogenization. Intron-exon boundaries were verified by aligning genomic and CDSs and confirming consensus splice site motifs (GT…AG for group I introns, variable for group II introns). Transfer RNA structures were validated using tRNAscan-SE v2.0 [15] with organellar search mode and a cove score cutoff of 15. Circular genome maps were generated using OGDRAW v1.3.1 [16]. Annotated sequences were deposited in GenBank under accession numbers NC_086516 (*V. molle*), NC_086692 (*V. dentatum*), NC_086693 (*V. tinus*), NC_086694 (*V. plicatum*), NC_086695 (*V. lentago*), NC_086696 (*V. amplificatum*), NC_086697 (*V. grandiflorum*), and PP101997 (*V. acerifolium*).

### 2.2. Comparative Sequence Analysis

Whole-plastome alignments were generated using MAFFT v7.505 [17] with the FFT-NS-i iterative refinement algorithm and default gap penalties. Sequence conservation was visualized using the mVISTA server [18] in Shuffle-LAGAN mode, which performs global alignment with automated detection of rearrangements, using *V. molle* as the reference sequence. Then, the DNA polymorphism module of DnaSP v6.12.03 [19] was used for nucleotide variability (*Pi*) analysis with a selected step size of 200 sites and a window length of 800 sites. This window size was selected to capture variation at the scale of individual genes while providing sufficient resolution to identify localized hotspots. Regions with *Pi* value exceeding 0.02 were flagged as hypervariable and evaluated as candidate molecular markers. Inverted repeat boundaries were compared across species using IRscope [20], which generates graphical representations of the four junction sites (JLB, JSB, JSA, JLA) showing gene positions relative to boundaries. This analysis identifies expansion or contraction events that alter the genes located at IR-SC junctions.

### 2.3. Repeat Sequence Identification

Simple sequence repeats (microsatellites) were identified using MISA v2.1 [21] with minimum repeat number thresholds of 10 for mononucleotides, 5 for dinucleotides, 4 for trinucleotides, and 3 for tetra-, penta-, and hexanucleotides. These thresholds were selected to balance sensitivity with specificity based on previous plastome SSR studies. Long dispersed repeats were identified using REPuter [22] with a minimum repeat size of 30 bp, maximum size of 300 bp, and Hamming distance of 3 (allowing for up to 3 mismatches per 30 bp). Four repeat types were detected: forward (direct), reverse, complement, and palindromic.

### 2.4. Codon Usage Analysis

Protein-coding sequences were extracted from each plastome, excluding genes with internal stop codons, frameshift mutations, or incomplete terminal codons. For genes with multiple copies (e.g., those in inverted repeats), a single representative copy was retained. Relative synonymous codon usage (RSCU) was calculated using CodonW v1.4.4 (http://codonw.sourceforge.net/ (accessed on 1 February 2026)). RSCU values were calculated as the observed frequency of a codon divided by the expected frequency under equal usage of synonymous alternatives; values greater than 1.0 indicate preferential usage. RSCU values were visualized as heatmaps using the pheatmap package in R v4.2.0, with hierarchical clustering based on Euclidean distances and complete linkage.

### 2.5. Selection Pressure Analysis

To ensure accurate codon alignment, protein-coding sequences across *Viburnum* species were analyzed with MACSE v2 [23]. The assessment of the ratio between non-synonymous (*Ka*) and synonymous (*Ks*) substitution rates was conducted using *Ka*/*Ks* Calculator v2 [24]. By comparing with *V. japonicum*, the *Ka*/*Ks* values for each *Viburnum* species were determined. Unrealistic *Ka*/*Ks* ratios were excluded to ensure precise screening of conserved and divergent genes. Thus, we adopted a more accurate threshold screening: *Ka*/*Ks* < 0.01 treated as qualified conserved genes, *Ka*/*Ks* = 1 for neutral selection, *Ka*/*Ks* >1 (greater than 1) as positively selected orthologs, and *Ka/Ks* < 1 (less than 1) for purifying selection.

### 2.6. Phylogenetic Analysis

For phylogenetic reconstruction, we assembled a dataset comprising the eight newly sequenced *Viburnum* plastomes, thirteen additional *Viburnum* species obtained from GenBank (representing major sections not covered by our sampling), and employed a hierarchical set of eighteen outgroup taxa spanning a range of phylogenetic distances to reconstruct the phylogeny. Specifically, closely related Caprifoliaceae taxa (*Sambucus* and *Lonicera*) were selected to stabilize internal branching patterns, moderately distant Dipsacales (*Dipelta* and *Linnaea*) lineages were used to calibrate key divergence nodes, and distantly related groups (*Lysimachia* and *Curtisia*) were included to root the tree reliably. This multi-layered approach ensures the robustness of the inferred phylogenetic topology. Complete plastome sequences were aligned using MAFFT v7.505 with the G-INS-i algorithm, which is optimized for global homology with accurate alignment of conserved regions. Ambiguously aligned regions were removed using trimAl v1.4 [25] with the automated1 heuristic, which selects trimming parameters based on alignment characteristics. The optimal partitioning scheme and substitution models were determined using ModelFinder [26] as implemented in IQ-TREE v2.2.0 [27], testing all models available in IQ-TREE and selecting based on the Bayesian Information Criterion (BIC). Bayesian inference was conducted using MrBayes v3.2.7 [28]. Two independent runs of four Metropolis-coupled MCMC chains (three heated, one cold) were run for 20 million generations, sampling every 1000 generations. The temperature parameter was set to 0.1 to improve chain mixing. Convergence was assessed by examining the average standard deviation of split frequencies (target < 0.01), potential scale reduction factors (target ≈ 1.0), and effective sample sizes (target > 200 for all parameters) using Tracer v1.7.2. The first 25% of samples were discarded as burn-in, and a 50% majority-rule consensus tree was constructed from the remaining samples.

## 3. Results

### 3.1. Plastome Structure and Gene Content

**Figure 1:**
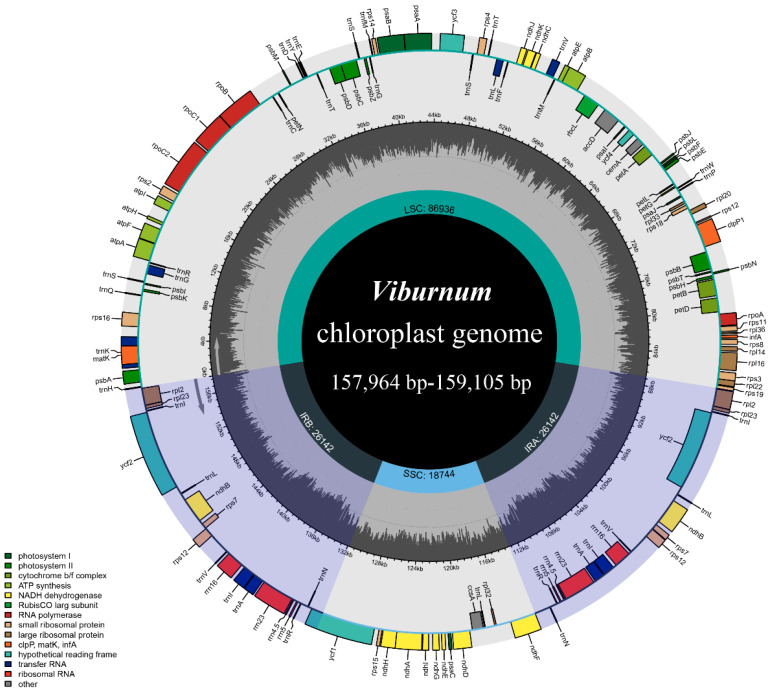
Circular map of chloroplast genomes in *Viburnum* plants. The inner gray ring is divided into four areas: SSC, IRb, LSC, and IRa. The genes in the outer ring region are transcribed clockwise, while those in the inner ring are transcribed counterclockwise. In addition, this figure also reflects the GC content, while the inner dark gray ring indicates the GC content.

Complete plastome assemblies were obtained for all eight *Viburnum* species, each displaying the canonical quadripartite structure characteristic of most photosynthetic angiosperms. Total plastome sizes ranged from 157,964 bp in *V. dentatum* to 159,015 bp in *V. amplificatum*, a difference of 1051 bp representing only 0.66% variation across the sampled species. This size variation was distributed across all four regions but was proportionally greatest in the small single-copy region, which ranged from 18,222 bp (*V. lentago*) to 18,744 bp (*V. dentatum*) a span of 522 bp (2.9%). The large single-copy region exhibited slightly less proportional variation (86,936–87,491 bp; 0.64%), while the inverted repeat regions were the most conserved in length (26,142–26,541 bp; 1.5%) (Table 1; Figure 1). These patterns suggest that size variation in *Viburnum* plastomes is driven primarily by indel accumulation in the single-copy regions rather than by large-scale expansion or contraction of the inverted repeats.

Overall GC content was remarkably uniform across species (38.1–38.2%) but showed the expected asymmetric distribution among regions. The inverted repeats exhibited the highest GC content (43.0–43.2%), attributable to the presence of GC-rich ribosomal RNA genes (*rrn16*, *rrn23*, *rrn4.5*, *rrn5*). The large single-copy region showed intermediate values (36.3–36.5%), while the small single-copy region had the lowest GC content (32.0–32.2%) (Table 1). This regional variation in nucleotide composition has implications for primer design in regions spanning multiple plastome compartments.

Gene content was highly conserved, with 129–131 genes annotated per plastome: 85–86 protein-coding genes, 36–37 tRNA genes, and 8 rRNA genes (Table 1). The minor variation in gene counts reflected differences in the annotation of boundary genes partially duplicated in inverted repeats and the status of putative pseudogenes. Eighteen genes contained introns: fifteen genes possessed a single intron (*atpF*, *ndhA*, *ndhB*, *petB*, *petD*, *rpl2*, *rpl16*, *rpoC1*, *rps16*, and six tRNA genes), while *clpP*, *ycf3*, and *rps12* each contained two introns. Seventeen genes were completely duplicated in the inverted repeats, including all four rRNA species, seven tRNAs, and six protein-coding genes (*ndhB*, *rpl2*, *rpl23*, *rps7*, *ycf2*, *rps12*) (Table S1).

### 3.2. Inverted Repeat Boundary Variation

**Figure 2:**
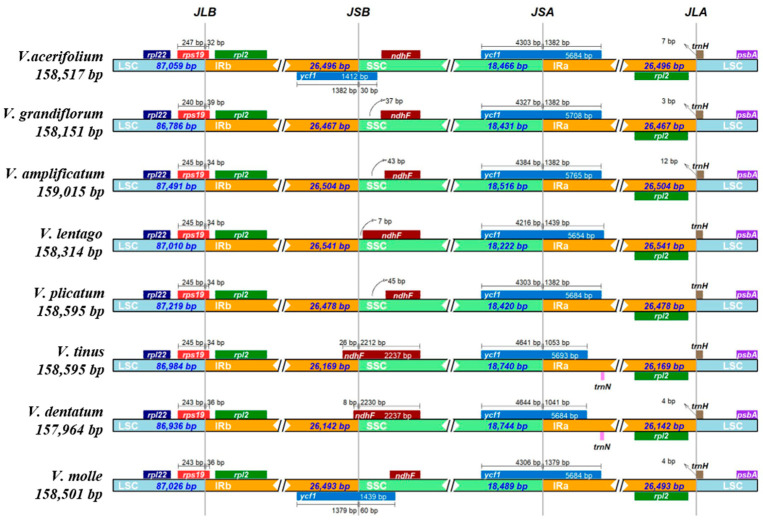
Comparison of junctions between the LSC, SSC, and IRs among eight species. JLB (IRb/LSC), JSB (IRb/SSC), JSA (SSC/IRa), and JLA (IRa/LSC). The number above indicates the distance in bp between the ends of the genes and the border sites (distances are not to scale in this figure).

Comparison of the four junctions between inverted repeat and single-copy regions revealed both conserved features and lineage-specific variations that illuminate microstructural evolution in *Viburnum* plastomes. At the JLB junction (LSC/IRb), *rpl22* was consistently positioned entirely within the large single-copy region across all species. The adjacent *rps19* gene showed partial duplication in all species, with 32–39 bp of its 3′ end extending into IRb (Figure 2).

The JSB junction (IRb/SSC) displayed more substantial variation. In six of the eight species, the *ndhF* gene was located entirely within the small single-copy region, with its 5′ end positioned 1–89 bp from the junction. However, in *V. tinus* and *V. dentatum*, *ndhF* extended 8–26 bp into IRb, indicating a minor expansion of the inverted repeat into the small single-copy region in these lineages. Notably, these two species also possessed the shortest inverted repeats in our sample (26,142 and 26,169 bp, respectively), suggesting that the *ndhF* boundary shift was accompanied by contraction elsewhere in the repeat. Additionally, *V. tinus* and *V. dentatum* harbored an extra *trnN-GUU* gene within IRa that was absent from the other six species, further supporting lineage-specific IR restructuring.

The JSA junction (SSC/IRa) was the most conserved boundary, with *ycf1* spanning the junction in all species. The larger 5′ portion of *ycf1* (4216–4644 bp) resided in SSC while the 3′ portion (1041–1439 bp) extended into IRa. This arrangement creates a truncated *ycf1* pseudogene at the JSB junction in IRb, ranging from 1000 to 1200 bp depending on species. The JLA junction (IRa/LSC) showed variable positioning of *trnH-GUG*: in *V. lentago*, *V. plicatum*, and *V. tinus*, this gene was located entirely within LSC (3–47 bp from the junction), while in the remaining species it extended 3–12 bp into IRa.

### 3.3. Sequence Divergence Patterns

**Figure 3:**
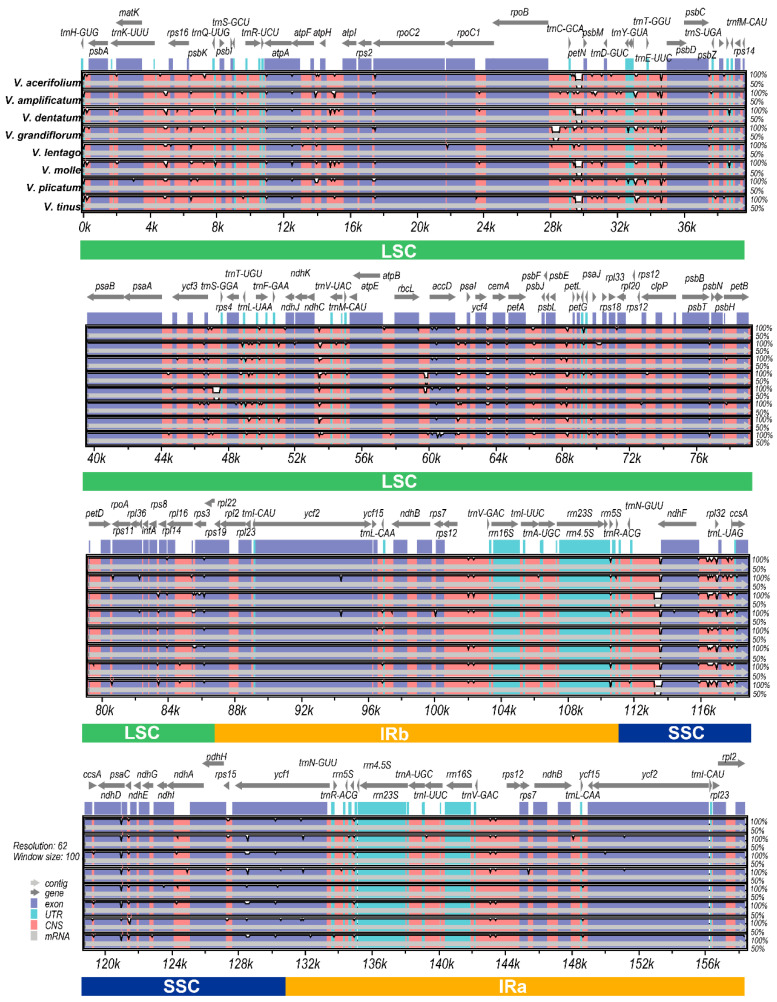
mVISTA comparison maps of chloroplast genomes of eight species of *Viburnum* plants. The x-axis indicates the coordinates in the chloroplast genome, and the y-axis represents the percentage identity from 50 to 100%. The gray arrows above indicated the extension direction of the gene, purple indicated the exon, blue indicated the untranslated region, pink indicated the non-coding sequences, and the grayish part indicated mRNA.

Global alignment of the eight plastomes revealed high overall sequence conservation punctuated by localized regions of elevated divergence. The mVISTA plot illustrated that sequence identity exceeded 90% across most of the alignment, with the inverted repeats showing particularly high conservation (>99% identity) as expected from their exposure to gene conversion. Divergence was concentrated in intergenic spacers and, to a lesser extent, in certain protein-coding genes, while ribosomal RNA and transfer RNA genes were nearly invariant (Figure 3).

**Figure 4:**
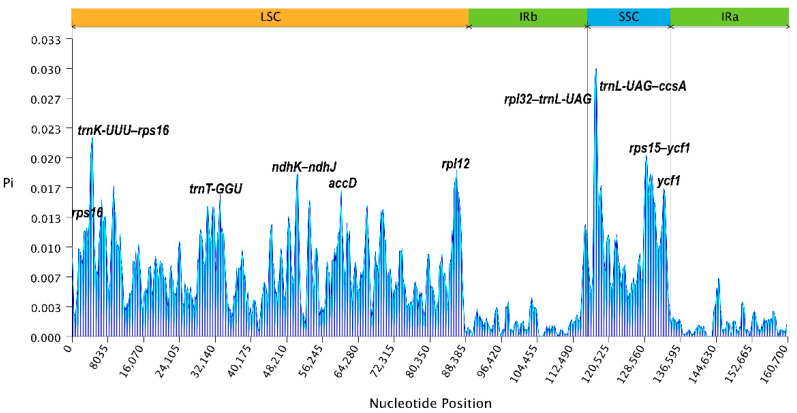
Nucleotide diversity (*Pi*) analysis of gene-coding regions and gene spacers in eight species. The abscissa represents the position, and the blue line represents the average of the nucleotide variations (*Pi*) of the eight species.

Sliding-window analysis quantified these patterns, revealing a genome-wide mean *Pi* value of 0.0059 (Figure 4). However, this average masked substantial regional heterogeneity: the small single-copy region showed the highest diversity (*Pi* value = 0.01), followed by the large single-copy region (*Pi* value = 0.0074), while the inverted repeats exhibited markedly lower values (*Pi* value = 0.0012). The 8.3-fold difference in *Pi* value between the IR and SSC regions reflects both the homogenizing effect of gene conversion on IR sequences and the concentration of rapidly evolving intergenic spacers in the single-copy regions.

The sliding-window analysis identified multiple peaks of elevated diversity distributed across the large single-copy region. The highest *Pi* value was observed in the *trnL-UAG–ccsA* intergenic spacer (*Pi* value = 0.0298). Other notably divergent regions included *rpl32–trnL-UAG* (*Pi* value = 0.0275), *trnK-UUU–rps16* (*Pi* value = 0.0222), and *rps15–ycf1* (*Pi* value = 0.0202). Among protein-coding genes, *rps16*, *ndhJ*, *ndhK*, *accD*, *rpl2*, *rps19*, *rpl22, ndhD*, *rps15*, *ndhH*, *psaC*, and *ycf1* showed elevated divergence relative to their functional categories, with *Pi* values ranging from 0.0160 to 0.0199.

### 3.4. Repeat Sequence Composition

**Figure 5:**
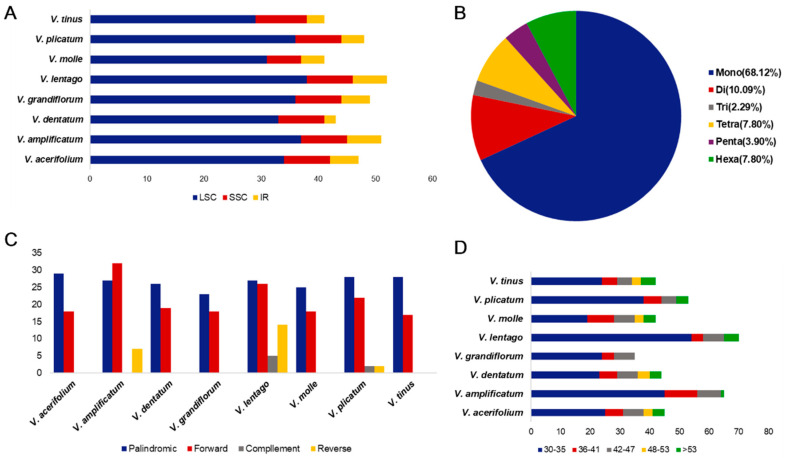
Analysis of simple sequence repeats (SSRs) and long repeats in the chloroplast genomes of *Viburnum* species. (**A**) Number of SSRs in LSC, SSC, and IR regions. (**B**) Type of shared SSRs among the chloroplast genomes. Number of SSRs in each species. mono-, mononucleotides; di-, dinucleotides; tri-, trinucleotides; tetra-, tetranucleotides; penta-, pentanucleotides; hexa-, hexanucleotides. (**C**) Number of long repeats by length. (**D**) Number of different long repeats.

Simple sequence repeat analysis identified 41–52 microsatellites per plastome, with *V. lentago* harboring the most and *V. molle* and *V. tinus* the fewest (Figure 5A). Mononucleotide repeats predominated, accounting for 68.12% of all SSRs across species and consisting almost exclusively of poly-A or poly-T tracts. This compositional bias reflects the AT-richness of plastome non-coding regions where most SSRs occur. Dinucleotide repeats comprised 10.09% of the total, all with AT/TA motifs, while tri-, tetra-, penta-, and hexanucleotide repeats accounted for the remaining 21.79% in approximately equal proportions. SSRs were non-randomly distributed across the plastome, with the large single-copy region harboring 70–76% of repeats, the small single-copy region containing 15–22%, and the inverted repeats holding only 4–12% (Figure 5B).

Long dispersed repeats (≥30 bp) numbered 41–72 per plastome, varying considerably among species. Forward and palindromic repeats together accounted for more than 73% of long repeats in all species, while reverse and complement repeats were comparatively rare (Figure 5C). The majority of long repeats (45–77%) fell within the 30–35 bp size class, with progressively fewer repeats at larger sizes. Repeats exceeding 50 bp were uncommon (less than 19% of the total) except for the inverted repeats themselves (Figure 5D). The functional significance of these dispersed repeats is unclear, but they may facilitate recombination-mediated rearrangements in some lineages.

### 3.5. Codon Usage Patterns

**Figure 6:**
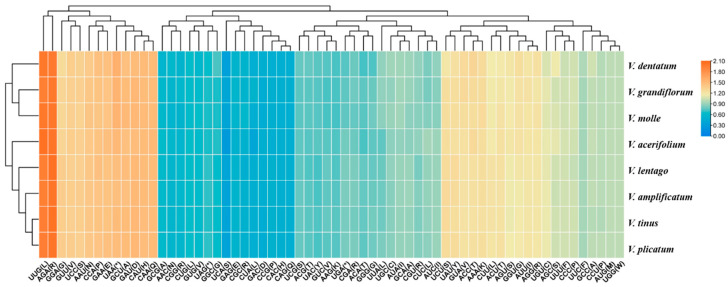
Heat map of relative codon preference (RSCU) analysis for *Viburnum* species. The RSCU values of 64 codons were used as the basis for tree clustering. As the red color deepens, the RSCU value increases. As the blue tint gets darker, the RSCU value decreases. Each column represents a different codon. Each row represents a distinct species of *Viburnum*. * indicates termination codons.

Relative synonymous codon usage (RSCU) analysis quantitatively confirmed a strong bias toward A- and T-ending synonymous codons. Of 61 sense codons (excluding the single codons for Met and Trp), 29 showed RSCU values exceeding 1.0, indicating preferential usage (Figure 6). Twenty-five of these twenty-nine preferred codons ended in A or U, while only two G-ending codons (AGG for Arg, UUG for Leu) showed preference. Three C-ending codons (AGC for Ser, CCC for Pro, GCC for Ala) exhibited slight preference. Conversely, 30 codons showed RSCU values below 1.0, predominantly those ending in C or G. The most strongly preferred codons were AGA (Arg; RSCU = 1.98–2.05), UUG (Leu; RSCU = 1.94–2.04), and GAA (Glu; RSCU = 1.44–1.48), while the most avoided codons were UCA (Ser; RSCU = 0.27–0.40), GAC (Asp; RSCU = 0.47–0.51), and CAG (Gln; RSCU = 0.48–0.50).

Hierarchical clustering based on RSCU values grouped the eight species into two clusters. One cluster contained *V. molle*, *V. dentatum*, and *V. grandiflorum*, while the second cluster comprised the remaining five species. The imperfect correspondence between codon usage similarity and phylogeny suggests that factors beyond shared ancestry—potentially including GC content variation, tRNA abundance, and translational selection—influence codon usage evolution in *Viburnum* plastomes.

### 3.6. Selection on Protein-Coding Genes

We calculated the *Ka*/*Ks* ratios for 78 protein-coding genes in its plastome using *V. japonicum* as the reference. Due to missing information (*Ks* = 0) preventing reliable determination for some genes, we chose to exclude these genes from the analysis. The majority of genes exhibited *Ka*/*Ks* ratios well below 1, indicating strong purifying selection acting on plastid protein-coding genes. Genes involved in photosynthesis showed particularly low *Ka*/*Ks* ratios, with many genes displaying values approaching zero. These included genes encoding photosystem I components (*psaA*, *psaB*, *psaC*, *psaJ*), photosystem II components (*psbA*, *psbB*, *psbC*, *psbD*), and ATP synthase subunits (*atpA*, *atpB*, *atpE*, *atpH*, *atpI*). In contrast, *accD*, *ycf1*, and *ycf2* exhibited the highest *Ka*/*Ks* ratios among all analyzed genes, although all values remained below 1 (Table S2). This indicates that these genomes have a higher degree of conservation.

### 3.7. Phylogenetic Relationships

**Figure 7:**
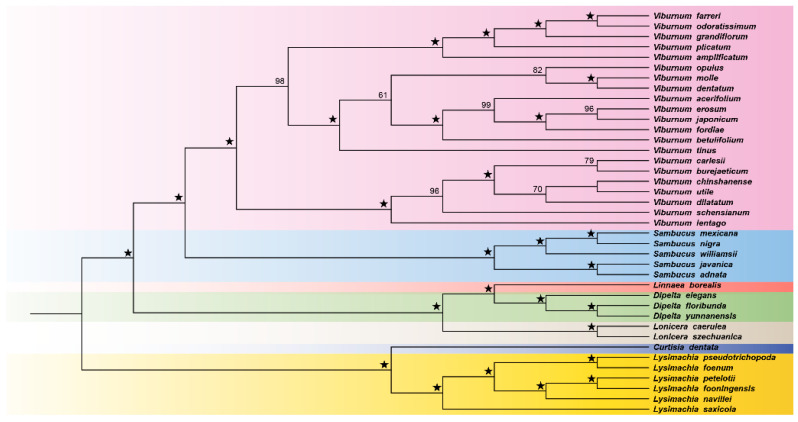
Phylogenetic trees constructed with the complete chloroplast genomes of *Viburnum* species using the Bayesian inference (BI) methods. *Lysimachia* and *Curtisia* were used as outgroups. Support values are indicated by the numbers above the nodes. ★ = 100.

Bayesian analyses produced identical topologies with strong support for most nodes. Within *Viburnum*, two major clades were recovered, each with maximum support (Bayesian posterior probability = 1.00). The first clade (Clade I) comprised seven species corresponding largely to species traditionally assigned to the sections Valvatotinus and Euviburnum. Within this clade, *V. lentago* was placed in the Lentago subclade of Valvatotinus, while *V. carlesii*, *V. burejaeticum*, *V. utile*, and *V. schensianum* formed a well-supported group corresponding to section Euviburnum. The relationships among Euviburnum species were fully resolved, with *V. carlesii* and *V. burejaeticum* as sister taxa (Figure 7).

The second major clade (Clade II) contained fourteen species from sections Solenotinus, Tomentosa, Crenotinus, Opulus, Porphyrotinus, Succotinus, and Tinus. This clade was subdivided into two sister groups. The first subgroup united members of Solenotinus, Tomentosa, and Crenotinus, with *V. grandiflorum* occupying a position at the base of Solenotinus, consistent with its basal morphological features within that section. Within this subgroup, *V. amplificatum* was determined to be a sister to the Crenotinus clade sensu stricto. The second subgroup comprised sections Opulus, Porphyrotinus, Succotinus, and Tinus. Within Porphyrotinus, *V. molle* and *V. dentatum* were placed in distinct subclades, and *V. tinus* occupied an isolated position within this group.

## 4. Discussion

### 4.1. Plastome Structural Conservation in the Context of Viburnum Diversification

The eight *Viburnum* plastomes characterized in this study exhibit remarkable structural conservation, with size variation of only 0.66% and identical gene content across species representing five sections and divergence times spanning the Oligocene to Miocene. This structural stasis contrasts with the extensive plastome rearrangements documented in some angiosperm lineages, such as Geraniaceae, where inversions, gene losses, and IR expansions have produced highly derived genome architectures [29]. The conservation observed in *Viburnum* likely reflects both the relatively recent crown age of the genus (~55 Ma) [30] and the strong functional constraints on plastome-encoded genes.

Although overall structure was conserved, we detected subtle variation at IR boundaries that illuminates ongoing microstructural evolution. The finding that *V. tinus* and *V. dentatum* share similar IR configurations, including *ndhF* extension into IRb and an additional *trnN-GUU* copy, despite belonging to different sections (Tinus and Porphyrotinus) presents an intriguing pattern. One interpretation is that this configuration represents the ancestral state for *Viburnum*, subsequently modified in other lineages. Alternatively, convergent boundary shifts may have occurred independently in these two species. Resolution of this question will require examination of IR boundaries across a broader sample of species, particularly those branching near *V. tinus* and *V. dentatum* in the phylogeny.

The regional heterogeneity in GC content observed in *Viburnum* plastomes—with IR regions substantially more GC-rich than single-copy regions—is a universal feature of angiosperm plastomes attributable to the presence of GC-rich ribosomal RNA genes in the IRs. However, the functional consequences of this compositional asymmetry remain incompletely understood. Recent studies have suggested that regional GC content may influence mutation rates, recombination frequencies, and gene expression levels, potentially contributing to the observed differences in evolutionary rates among plastome regions [31].

### 4.2. Hypervariable Regions and Their Utility as Molecular Markers

The identification of eleven intergenic regions with nucleotide diversity exceeding 0.02 provides a robust set of candidate markers for various applications in *Viburnum* research. Several of these regions, including *trnK-UUU–rps16*, *rpl32–trnL-UAG*, and *trnL-UAG–ccsA*, have been previously identified as phylogenetically informative in Dipsacales and other angiosperm families [32,33], supporting their general utility. The *petN–trnC-GCA* spacer is particularly promising because it combines high variability with conserved flanking regions that facilitate primer design, and it has been successfully employed for species discrimination in several plant genera.

For practical applications, we suggest a tiered approach to marker selection depending on the taxonomic level of interest. For discrimination among closely related species or infraspecific taxa, the most variable regions (*trnK-UUU–rps16*, *trnL-UAG–ccsA*) offer maximum resolution. For genus-wide phylogenetics, moderately variable regions with fewer alignment difficulties are more appropriate. For DNA barcoding applications requiring standardized markers, *ycf1* emerges as a strong candidate: it combines high variability with a length suitable for single-amplicon sequencing and has been proposed as a universal barcode for land plants [34].

The predominance of mononucleotide A/T repeats is typical of plastome SSRs and facilitates primer design in flanking regions. However, mononucleotide repeats can be challenging to score accurately due to polymerase slippage during PCR, and di- or trinucleotide repeats may be preferable for population genetic applications where precise allele calling is essential.

### 4.3. Relaxed Selection on Non-Photosynthetic Genes

The finding that *accD*, *ycf1*, and *ycf2* evolve under relaxed purifying selection relative to photosynthesis genes is consistent with observations across diverse angiosperm lineages and illuminates the different selective regimes experienced by plastome genes with distinct functions [35]. Photosynthesis genes encode proteins that must function within highly integrated complexes where individual subunits interact with multiple partners; amino acid changes that disrupt these interactions are deleterious and eliminated by selection. In contrast, genes involved in other processes may experience weaker constraint, particularly if their functions can be partially complemented by nuclear-encoded paralogs or if the processes they mediate are themselves subject to adaptive modification.

The elevated *Ka*/*Ks* of *accD* is particularly noteworthy because this gene has been lost from plastomes in multiple angiosperm lineages, including grasses, with its function assumed by nuclear-encoded acetyl-CoA carboxylase [36]. In *Viburnum*, *accD* remains intact and functional, but its relaxed constraint may indicate incipient functional transfer or simply reduced selection intensity in a genus where fatty acid metabolism varies among species with different ecological strategies. The observation that *accD* is also among the most sequence-divergent protein-coding genes suggests that its elevated *Ka*/*Ks* translates into substantial sequence variation useful for phylogenetic and barcoding applications.

The relaxed evolution of *ycf1* and *ycf2* reflects the coevolutionary dynamics inherent in protein import systems. These genes encode components of the TIC translocon, which must recognize and translocate thousands of different nuclear-encoded proteins with diverse transit peptides [37]. Maintaining this broad substrate specificity while preserving transport fidelity may require a degree of sequence flexibility not permitted in the more structurally constrained photosynthesis complexes. Consistent with this interpretation, *ycf1* and *ycf2* show elevated *Ka*/*Ks* across most angiosperm lineages examined, suggesting that relaxed constraint on protein import genes is a general feature of plastome evolution rather than a *Viburnum*-specific phenomenon.

### 4.4. Phylogenetic Implications

The phylogenetic relationships recovered in this study are highly congruent with previous molecular systematic analyses of *Viburnum* [6,8], confirming the robustness of plastome data for resolving infrageneric relationships. The recognition of two major clades—one comprising sections Valvatotinus and Euviburnum, the other containing the remaining sections—appears to represent a fundamental division within the genus that may correspond to an ancient biogeographic or ecological divergence [30]. We inferred that this split occurred in the Eocene, with subsequent diversification in each clade associated with distinct geographic regions and climatic niches.

Two specific results merit discussion in the context of taxonomic revision. First, the placement of *V. amplificatum* as sister to Crenotinus sensu stricto, rather than within it, suggests that this Southeast Asian species may warrant recognition as a distinct section or subsection. Its intermediate morphological features and isolated phylogenetic position indicate a unique evolutionary trajectory that would be obscured by subsuming it within Crenotinus. Second, the isolated position of *V. tinus* within Subclade IIb reinforces its distinctiveness as the sole representative of the section Tinus. This Mediterranean endemic possesses a combination of characters—including evergreen habit, coriaceous leaves, and metallic blue drupes—found in no other *Viburnum* species, consistent with a long period of independent evolution in a geographically isolated region [1].

The relationships among North American species are also clarified by our analysis. The separation of *V. molle* (Mollotinus) from *V. dentatum* (Dentata) within section Porphyrotinus, and their collective distinction from *V. acerifolium* (Succotinus), confirms that these eastern North American species represent at least two independent colonization events from Asian ancestors, as inferred by Spriggs et al. (2015) [38]. This biogeographic pattern of multiple trans-Beringian dispersals is consistent with the genus’s center of diversity in Asia and the relatively recent (Miocene–Pliocene) establishment of *Viburnum* in the Americas.

## 5. Conclusions

Comparative analysis of eight *Viburnum* plastomes has revealed a structurally conserved genome harboring substantial sequence variation suitable for diverse molecular applications. The identification of eleven hypervariable intergenic regions and the characterization of selection pressures across protein-coding genes provide resources for population genetics, phylogeography, and species identification. The finding that genes involved in fatty acid biosynthesis (*accD*) and protein import (*ycf1*, *ycf2*) evolve under relaxed constraints relative to photosynthesis genes suggests that these loci experience distinct selective regimes potentially linked to functional divergence or coevolution with nuclear partners. Phylogenetic analysis confirms established sectional relationships while providing an improved resolution for taxonomically problematic taxa, including *V. amplificatum* and *V. tinus*. These results supplement the chloroplast genome database of the genus *Viburnum* and contribute to understanding plastome evolution in this ecologically important genus.

## Figures and Tables

**Table 1:** Chloroplast genomic characteristics of eight species within the genus *Viburnum*.

Genome Features	*V. molle*	*V. dentatum*	*V. acerifolium*	*V. amplificatum*	*V. grandiflorum*	*V. tinus*	*V. plicatum*	*V. lentago*
Genome size (bp)	158,501	157,964	158,517	159,015	158,151	158,062	158,595	158,314
LSC size (bp)	87,026	86,936	87,059	87,491	86,786	86,984	87,219	87,010
SSC size (bp)	18,489	18,744	18,466	18,516	18,431	18,740	18,420	18,222
IR size (bp)	26,493	26,142	26,496	26,504	26,467	26,169	26,478	26,541
Total GC content (%)	38.2	38.2	38.1	38.1	38.1	38.1	38.1	38.1
GC content in LSC (%)	36.5	36.5	36.4	36.3	36.4	36.4	36.3	36.3
GC content in SSC (%)	32.1	32.2	32.0	32.1	32.0	32.0	32.0	32.0
GC content in IR (%)	43.0	43.2	43.0	43.0	43.0	43.1	43.0	43.0
Number of genes	131	130	129	130	130	130	130	130
Protein genes	86	85	85	85	85	85	85	85
tRNA genes	37	37	36	37	37	37	37	37
rRNA genes	8	8	8	8	8	8	8	8

## Data Availability

Annotated plastome sequences are available in GenBank under accession numbers NC_086516, NC_086692, NC_086693, NC_086694, NC_086695, NC_086696, NC_086697, and PP101997.

## Supplementary Materials

The following supporting information can be downloaded at: https://www.mdpi.com/article/10.3390/genes17020196/s1, Table S1. Genes in the chloroplast genome of *Viburnum* species. Table S2: *Ka*/*Ks* of chloroplast genes regions in *Viburnum.*
