# Supplementary material for: Plastome Evolution in Viburnum (Adoxaceae): Comparative Genomics Reveals Hypervariable Markers and Relaxed Selection on Protein Import Genes
Source: Genes (Basel). 2026 Feb 6;17(2):196. doi: 10.3390/genes17020196 (PMC12941271; doi:10.3390/genes17020196)
Supplement: Supplementary file 1 [file genes-17-00196-s001.zip › genes-4139165-supplementary.pdf]

Table S1. Genes in the chloroplast genome of *Viburnum* species

Category	Gene group	Gene name
**Photosynthesis**	Subunits of photosystem I	*psaA, psaB, psaC, psaI, psaJ*
	Subunits of photosystem II	*psbA, psbB, psbC, psbD, psbE, psbF, psbH, psbI, psbJ, psbK, psbL, psbM, psbT, psbZ*
	Subunits of NADH dehydrogenase	*ndhA*(2), ndhB*(2), ndhC, ndhD, ndhE, ndhF, ndhG, ndhH, ndhI, ndhJ, ndhK*
	Subunits of cytochrome b/f complex	*petA, petB*, petD*, petG, petL, petN*
	Subunits of ATP synthase	*atpA, atpB, atpE, atpF*(2), atpH, atpI*
	Large subunit of rubisco	*rbcL*
	Subunits photochlorophyllide reductase	-
**Self-replication**	Proteins of large ribosomal subunit	*rpl14, rpl16*, rpl2*(2), rpl20, rpl22, rpl23(2), rpl32, rpl33, rpl36*
	Proteins of small ribosomal subunit	*rps11, rps12**(4), rps14, rps15, rps16*, rps18, rps19, rps19-fragment, rps2, rps3, rps4, rps7(2), rps8*
	Subunits of RNA polymerase	*rpoA, rpoB, rpoC1*, rpoC2*
	Ribosomal RNAs	*rrn16(2), rrn23(4), rrn4.5(2), rrn5(2)*
	Transfer RNAs	*trnA-UGC*(2), trnC-GCA, trnD-GUC, trnE-UUC, trnF-GAA, trnG-GCC, trnG-UCC*, trnH-GUG, trnI-CAU(2), trnI-GAU*(2), trnK-UUU*, trnL-CAA(2), trnL-UAA*, trnL-UAG, trnM-CAU, trnN-GUU(2), trnP-UGG, trnQ-UUG, trnR-ACG(2), trnR-UCU, trnS-GCU, trnS-GGA, trnS-UGA, trnT-GGU, trnT-UGU, trnV-GAC(2), trnV-UAC*, trnW-CCA, trnY-GUA, trnfM-CAU*
**Other genes**	Maturase	*matK*
	Protease	*clpP1***
	Envelope membrane protein	*cemA*
	Acetyl-CoA carboxylase	*accD*
	c-type cytochrome synthesis gene	*ccsA*
	Translation initiation factor	*infA*
	other	*pafI**(2), pafII, pbf1*
**Genes of unknown function**	Conserved hypothetical chloroplast ORF	*ycf1(2), ycf2(2)*

Notes: Gene*: gene with one introns; Gene**: gene with two introns; Gene (2): number of copies of multi-copy genes.

Table S2. ***Ka/Ks*** **of chloroplast genes regions in** ***Viburnum***

Gene	*V. molle*	*V. dentatum*	*V. tinus*	*V. plicatum*	*V. lentago*	*V. amplificatum*	*V. grandiflorum*	*V. acerifolium*
accD	0.809254	0.603431	0.925843	1.98617	1.53854	2.50083	1.15525	0.925464
atpA	0.258019	0.186772	0.0378533	0.0803103	0.0935372	0.161509	0.0923303	0
atpB	0.146564	0.309129	0	0.337411	0.534269	0.543102	0.115266	0.268199
ccsA	0.285109	0.209299	0.205863	0.197061	0.1777	0.163838	0.135035	0
infA	0	0	0.218476	0	0	0	0	0
ndhA	0	0	0	0.373459	0.0891359	0.449239	0.169996	0
ndhD	0	0.139002	0.188831	0.245763	0.0869774	0.20637	0.189717	0.46688
ndhF	0.237087	0.272156	0.507745	0.37658	0.442987	0.441321	0.492368	0.194986
ndhG	0	0.182989	0.237162	0.118669	0.313217	0	0	0
ndhI	0	0	0	0.0473003	0.0642325	0.0473003	0.277614	0
ndhJ	0	0	0	0	0	0	0	0
petA	0.10683	0.0964534	0.184049	0.181982	0.103057	0.227776	0.182531	0.30674
petD	0.0894387	0.0665137	0.198449	0.198449	0.198449	0.198449	0.111566	0.335597
psaA	0.137444	0.0531228	0.325275	0.176855	0.263896	0.187375	0.214252	0
psaJ	0	0	0	0	0	0	0	0
psbC	0.129736	0.259891	0.134963	0.531042	0.110031	0	0.661058	0.258625
